# The challenge of molecular selection in liver-limited metastatic colorectal cancer for surgical resection: a systematic review and meta-analysis in the context of current and future approaches

**DOI:** 10.32604/or.2024.049181

**Published:** 2024-08-23

**Authors:** ROSSANA RONCATO, JERRY POLESEL, FEDERICA TOSI, ELENA PERUZZI, ERIKA BRUGUGNOLI, CLAUDIA LAURIA PANTANO, MARIA FURFARO, FILIPPO DI GIROLAMO, ALESSANDRO NANI, ARIANNA PANI, NOEMI MILAN, ELENA DE MATTIA, ANDREA SARTORE-BIANCHI, ERIKA CECCHIN

**Affiliations:** 1 Experimental and Clinical Pharmacology Unit, Centro di Riferimento Oncologico di Aviano (CRO) IRCCS, Aviano, 33081, Italy; 2 Department of Medicine (DMED), University of Udine, Udine, 33100, Italy; 3 Unit of Cancer Epidemiology, Centro di Riferimento Oncologico di Aviano (CRO) IRCCS, Aviano, 33081, Italy; 4 Department of Oncology and Hemato-Oncology, Università degli Studi di Milano, Milan, 20122, Italy; 5 Department of Hematology, Oncology, and Molecular Medicine, Grande Ospedale Metropolitano Niguarda, Milan, 20122, Italy; 6 Oncology Pharmacy Unit, IRCCS Istituto Romagnolo per lo Studio dei Tumori Dino Amadori, Meldola, 47014, Italy; 7 Pharmacy Unit, Fondazione IRCCS Istituto Tumori di Milano, Milan, 20122, Italy; 8 Department of Pharmacy, Ca’ Foncello Treviso Regional Hospital, Piazzale Ospedale 1, Treviso, 31100, Italy; 9 Department of Medical Surgical and Health Sciences, University of Trieste, Trieste, 34127, Italy; 10 Hospital Pharmacy, Cattinara Hospital, Azienda Sanitaria Universitaria Giuliano Isontina, Trieste, 34148, Italy; 11 Department of Oncology and Onco-Hematology, Postgraduate School of Clinical Pharmacology and Toxicology, University of Milan, Milan, 20122, Italy

**Keywords:** Metastasectomy, Liver metastases, RAS, BRAF, SMAD4, PIK3CA, Colorectal

## Abstract

**Objectives:**

Treatment of metastatic colorectal cancer (mCRC) includes resection of liver metastases (LM), however, no validated biomarker identifies patients most likely to benefit from this procedure. This meta-analysis aimed to assess the impact of the most relevant molecular alterations in cancer-related genes of CRC (i.e., RAS, BRAF, SMAD4, PIK3CA) as prognostic markers of survival and disease recurrence in patients with mCRC surgically treated by LM resection.

**Methods:**

A systematic literature review was performed to identify studies reporting data regarding survival and/or recurrence in patients that underwent complete liver resection for CRC LM, stratified according to RAS, BRAF, PIK3CA, and SMAD4 mutational status. Hazard ratios (HRs) from multivariate analyses were pooled in the meta-analysis and various adjustment strategies for confounding factors were combined. The search was conducted in numerous databases, including MEDLINE (PubMed), Embase, Cumulative Index to Nursing and Allied Health Literature (CINAHL) (EBSCO host), and WHO Global Index Medicus, through March 18th, 2022. Meta-analyses, editorials, letters to the editor, case reports, studies on other primary cancers, studies with primary metastatic sites other than the liver, studies lacking specific oncological outcome variables or genetic data, non-English language studies, and studies omitting residual disease data from liver metastasectomy were excluded. The remaining 47 studies were summarized in a descriptive table which outlines the key characteristics of each study and final results were graphically presented.

**Results:**

RAS mutation status was negatively associated with overall survival (OS) (HR, 1.68; 95% CI, 1.54–1.84) and recurrence free survival (RFS) (HR, 1.46; 95% CI, 1.33–1.61). A negative association was also found for BRAF regarding OS (HR, 2.64; 95% CI, 2.15–3.24) and RFS (HR, 1.89; 95% CI, 1.32–2.73) and SMAD4 regarding OS (HR, 1.93; 95% CI, 1.56–2.38) and RFS (HR, 1.95; 95% CI, 1.31–2.91). For PIK3CA only three studies were eligible and no significant association with either OS or RFS could be highlighted.

**Conclusion:**

RAS, BRAF, and SMAD4 are negatively associated with OS and RFS in patients undergoing curative liver metastasectomy from colorectal cancer. No conclusion can be drawn for PIK3CA due to the limited literature availability. These data support the integration of RAS, BRAF, and SMAD4 mutational status in the surgical decision-making for colorectal liver metastasis. Nevertheless, we have to consider several limitations, the major ones being the pooling of results from studies that evaluated patient outcomes as either disease-free survival (DFS) or RFS; the inclusion of patients with minimal residual disease and unconsidered potential confounding factors, such as variability in resectability definitions, chemotherapy use, and a potential interaction between biological markers and pre- and post-resection pharmacological treatments.

## Contents:

- Only a small percentage of colorectal cancer patients benefits from surgical liver metastasis resection.
- This meta-analysis was performed with the aim to elucidate the role of previously investigated molecular prognostic factors that may help the surgical decision making for colorectal liver metastasis.
- The association between the mutational status of RAS, BRAF, SMAD4 and PIK3CA and overall survival (OS) and recurrence free survival (RFS) was evaluated in the 47 studies deemed as eligible.

## Introduction

Colorectal cancer (CRC) representing 9.7% of global cancer incidence, stands as the third most prevalent cancer type. Often diagnosed in advanced stages, approximately 75% of colon cancers are initially identified as local disease, amenable to surgical intervention [1].

Adjuvant chemotherapy in stage II and III tumors has shown an absolute survival benefit of up to 5% and 20%, respectively [2]. The liver is the primary site for metastases, with about 30% of CRC patients either presenting with or developing liver metastases (LM) during their disease course. The advent of novel targeted agents, a deeper understanding of molecular tumor biology, and the development of precision medicine have markedly improved the life expectancy of patients with advanced CRC, now averaging 40 months in certain subgroups [3–5]. Despite these advances, distant metastases remain the leading cause of mortality in CRC.

The most effective strategy to enhance prognosis in these cases is surgical resection of liver metastases, which can yield a 5-year overall survival (OS) rate of up to 55% when performed radically. Criteria for considering a patient for metastasectomy include the feasibility of complete metastasis removal with clear margins and sufficient residual liver volume [5]. However, only about 20% of LMs are deemed resectable at diagnosis.

Pre-operative or ‘conversion’ chemotherapy, employing cytotoxic agents like fluoropyrimidines with oxaliplatin and/or irinotecan, alone or in combination with targeted agents such as bevacizumab or cetuximab or panitumumab, serve varied purposes. Perioperative treatment assesses tumor response and conserves hepatic tissue for curative resection, while conversion therapy aims to render initially unresectable or borderline resectable LM surgically treatable. Advances in chemotherapy and perioperative care have broadened the patient pool for hepatic resection [6,7]. Nevertheless, post-resection disease recurrence, impacting 50%–75% of patients within five years, significantly affects survival [7].

Traditionally, clinical criteria like Fong’s clinical score and various nomograms have been used to predict outcomes post-LM resection. These consider factors like tumor size, lymph node invasion, number of metastases, and patient demographics [8]. Recently, molecular data, especially with pre-operative targeted therapy based on tumor mutational status, are being increasingly considered for ‘precision surgery’ in CRC LM [9].

Over the past decade, biomarkers such as *KRAS, NRAS* and *BRAF* have become crucial for the management of metastatic CRC. Notably, about 40% of patients diagnosed with metastatic CRC harbor mutations in exon 2 of *KRAS* codons 12 and 13 [10,11]. Additionally, approximately 5% have mutations in exons 3 or 4 of *KRAS* codons 61 or 146, and another 5% exhibit mutations in exons 2, 3, or 4 of *NRAS* [10,11]. Significantly, mutations affecting *BRAF* codon 600 are found in 10% of patients, with the V600E mutation specifically identified as a marker of poor prognosis [12,13].

The presence of RAS mutations, particularly in the metastatic context, is a well-established negative predictive biomarker for the efficacy of anti-EGFR drug [14,15]. Similarly, *BRAF* mutation, especially the V600E variant, has been shown to diminish the efficacy of anti-EGFR agents [16]. Moreover, both *BRAF* and *RAS* mutations are associated with a poorer prognosis in advanced disease [17,18].

Emerging evidence underscores the importance of pre-treatment identification of patients who are likely to benefit from liver metastasectomy. Studies have linked *KRAS* and *NRAS* mutations with reduced OS and recurrence-free survival (RFS) following hepatic metastasis resection. *BRAF* mutations, which occur in about 3% of patients with resected LM, demonstrate a similar impact. Additionally, somatic mutations in other relevant cancer genes, such as *TP53* and *SMADs*, have been explored, either individually or in combination, for their potential to stratify patients’ prognoses post-LM resection in CRC [7,19,20].

The aims of this review are to: 1) systematically analyze current literature and perform a meta-analysis of the available data on the impact of key genetic variants in CRC-related genes (i.e., *KRAS, NRAS, BRAF, SMAD4, PIK3CA*) as prognostic markers in surgically treated metastatic CRC patients; and 2) explore the potential role of emerging biomarkers like HER2 and microsatellite instability (MSI) status in current and novel therapeutic strategies, including liver transplantation for LM.

## Material and Methods

### Search strategy

**Figure 1:**
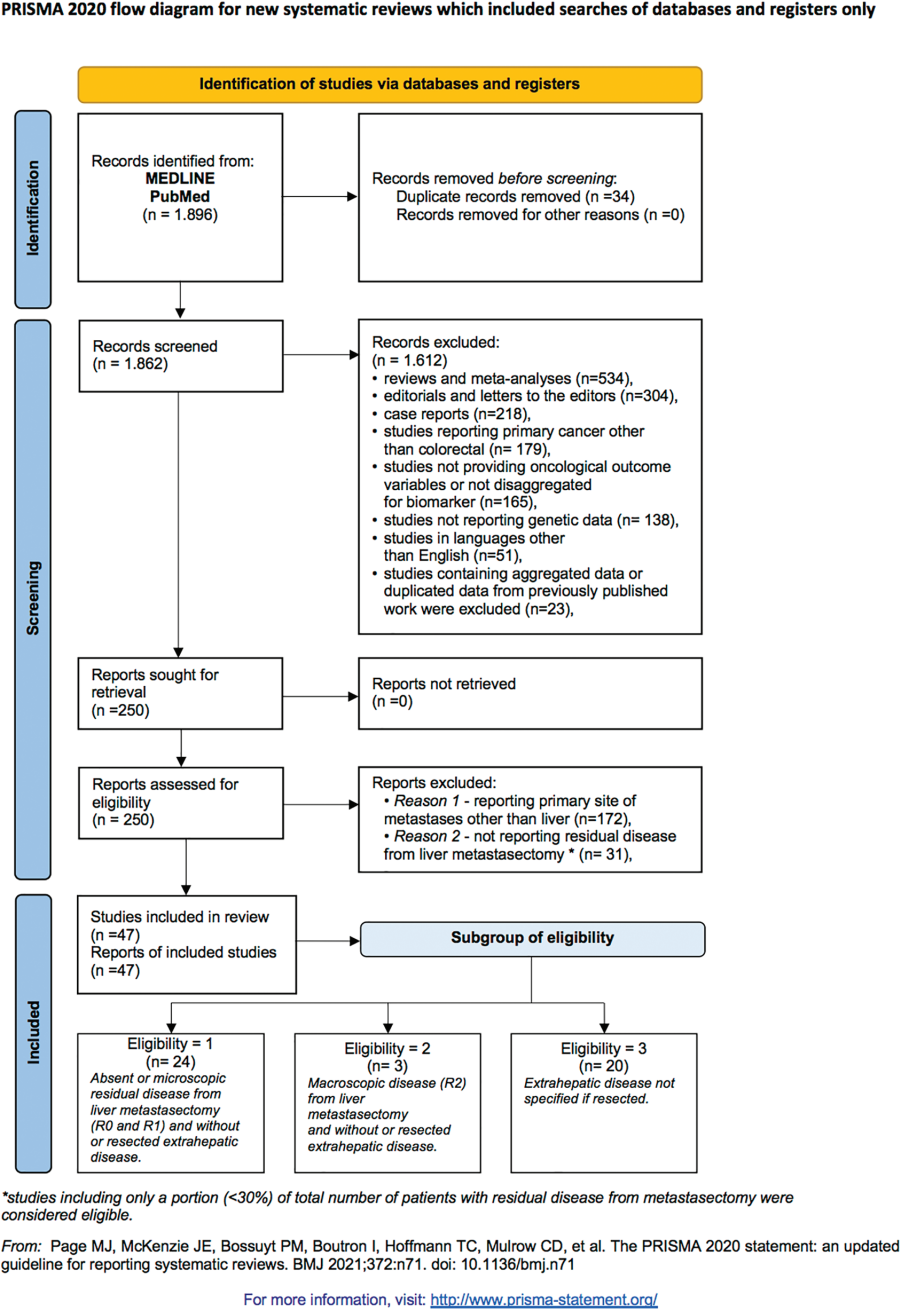
Article screening and selection according to preferred reporting items for systematic reviews and meta-analyses 2020 criteria flow diagram.

A systematic review was conducted by searching databases including MEDLINE (Pubmed), Embase, Cumulative Index to Nursing and Allied Health Literature (CINAHL) (EBSCO host), and WHO Global Index Medicus, through March 18th, 2022. The search algorithm used was: “(‘BRAF’ OR ‘RAS’ OR ‘HER2’ ‘SMAD4’ OR ‘TP53’ OR ‘P53’ OR ‘APC’ OR ‘PI3K’ OR ‘EGFR’ OR ‘MACC1’) and (‘colon’ or ‘colorectal’ or ‘rectal’ or ‘rectum’) and (‘metastasis’ or ‘metastatic’ or ‘metastases’ or ‘mets’ or ‘metastasectomy’) and (‘hepatic’ or ‘liver’)”. Titles and abstracts of identified records were screened for relevance. Since MEDLINE included all the articles returned by the other two databases, we referred only to MEDLINE in Fig. 1.

### Selection criteria

Eligible studies included those detailing tumor mutation status and oncological outcomes in CRC patients who underwent liver metastasectomy. The inclusion criteria comprised studies performed in metastatic CRC patients undergoing liver metastases resection; reporting genetic test results for *RAS*, *BRAF*, *SMAD4*, or *PIK3CA* mutation status from resected colorectal liver metastasis (CRLM) specimens; providing outcomes of RFS and/or OS based on mutational status; and offering RFS and OS hazard ratios (HR) from multivariate analyses.

Exclusion criteria included reviews, meta-analyses, editorials, letters to the editor, case reports, studies on primary cancers other than colorectal, studies with primary metastatic sites other than liver, studies lacking specific oncological outcome variables or genetic data, non-English language studies, and studies omitting residual disease data from liver metastasectomy.

Three subgroups of eligibility were identified: “group 1” included studies with no or microscopic post-metastasectomy residual disease (R0 and R1) and without or resected extrahepatic disease; “group 2” encompassed studies with macroscopic post-metastasectomy residual disease (R2) and without or resected extrahepatic disease; “group 3” comprised studies where the status of extrahepatic disease was unspecified (either resected or not).

### Data extraction and statistical analysis

A predefined protocol was employed for data extraction. Extracted variables included: first author’s name, publication year, journal name, enrollment years, country of enrollment, sample size, study design, demographic profile, genes analyzed, genotyping method, tumor mutational status, chemotherapy scheme and schedule, percentage of patients with residual disease post-metastasectomy, percentage with extra-hepatic disease and oncological outcomes (OS, overall response rate (ORR), disease-free-survival (DFS), or RFS) + including main findings and 95% confidence interval (CIs). Four authors (MF, FDG, CLP, EC) independently verified inclusion criteria and executed data extraction. Adjusted estimates from original studies for relevant confounding factors were utilized.

The standard error of the log HR was deduced from the log CIs. Pooled HR and corresponding 95% CI were calculated using the DerSimonian and Laird random-effects models, accounting for within-study and between-study variabilities. In cases of a low number of studies, pooled HR was also estimated via the Hartung-Knapp-Sidik-Jonkman (HKSJ) method for a conservative approach. Statistical heterogeneity among studies was assessed using the I² and Q statistics. Influence analysis was conducted, recalculating pooled HR by sequentially omitting each study. Publication bias was evaluated through funnel plot.

Meta-analysis results were graphically presented, depicting HRs as black squares (size inversely proportional to standard error) and 95% CIs. In some studies, 95% CIs were slightly modified from those in original papers due to estimated study variances. Pooled HRs for all studies and various study designs were represented by diamonds, indicating the HR at the center and 95% CIs at the extremes. Statistical significance was set at *p* < 0.05 (two-sided).

## Results

We included 47 studies that evaluated the predictive value of the somatic genetic profile for outcomes following liver metastasectomy in CRC patients (Fig. 1) [8,20,21–65]. Our analysis was limited to genetic testing data for *RAS* or *BRAF* or *SMAD4* or *PI3KA* mutations due to insufficient data for other biomarkers. Insufficient study numbers precluded meta-analysis for HER2 and MSI status; however, we described the main evidence available. A descriptive table outlines the key characteristics of studies included in the meta-analysis (Table 1).

**Table 1:** Medical treatment administered in the studies included in the meta-analysis

First author	Year	Region	No. of patients	*KRAS*	*BRAF*	*PIK3CA*	*SMAD4*	Endpoint	CT before resection only	CT After resection only	CT before and after resection	Regimen
Kawaguchi et al. [32]	2021a	USA	561	*RAS (KRAS/NRAS)*	*BRAF*	*PIK3CA*	*SMAD4*	OS	90,3%	NA	NA	NA
Kawaguchi et al. [33]	2021b	USA	790	*RAS (KRAS/NRAS)*				OS	88,2%	NA	NA	NA
Hoppener et al. [29]	2021	Netherlands and USA	780	*KRAS*	*BRAF*			OS, RFS	0,0%	0,0%	0,0%	NA
Liu et al. [40]	2021	China	769	*RAS (KRAS/NRAS)*				RFS	61,0%	79,2%	NA	5-FU with OXA/IRI with or without anti-EGFR or anti-VEGF
Nishioka et al. [47]	2021	USA	552		*BRAF*		*SMAD4*	RFS	87,0%	74,0%	NA	5-FU with OXA/IRI with or without anti-EGFR or anti-VEGF
Takeda et al. [62]	2021a	Japan	409	*KRAS*				OS	44,5%	0,0%	0,0%	NA
Takeda et al. [63]	2021b	Japan	341	*RAS (KRAS/NRAS)*				OS	52,0%	56,0%	ND	NA
Margonis et al. [45]	2021	USA (Baltimore)	718	*KRAS*				OS	65,1%	57,0%	NA	NA
Kawaguchi et al. [35]	2020a	USA	476	*RAS (KRAS/NRAS)*	*BRAF*		*SMAD4*	OS, RFS	90.5%	NA	NA	NA
Kawaguchi et al. [34]	2020b	USA	416	*KRAS*				RFS	76,0%	NA	NA	NA
Baldin et al. [24]	2020	Belgium	221	*RAS (KRAS/NRAS)*				OS, RFS	84,6%	NA	NA	5-FU with OXA/IRI with or without anti-EGFR or anti-VEGF
Brunsell et al. [27]	2020	Norway	106			*PIK3CA*		OS	82,0%	NA	NA	5-FU with OXA and/or IRI with anti-EGFR and/or anti-VEGF
Gholami et al. [28]	2020	USA	487	*RAS (KRAS/NRAS)*				OS, RFS	42,4%	100,0%	42,4%	Adjuvant HAI pump with 5-FU (56%) and/or systemic 5-FU
Jàcome et al. [31]	2020	USA (Texas)	573	*RAS (KRAS/NRAS)*	*BRAF*			OS	87,0%	69,0%	NA	NA (oxaliplatin-based regimen plus or minus BEVA)
Saadat et al. [51]	2020	USA	938	*RAS (KRAS/NRAS)*				OS, RFS	64,1%	20,4%	NA	NA
Allievi et al. [21]	2019	Italy and USA	806	*KRAS*				OS	NA	NA	NA	NA
Bachet et al. [23]	2019	France	249		*BRAF*			OS, RFS	82,7%	74,7%	NA	5-FU with OXA and targeted agent
Brudvik et al. [25]	2019	USA (Texas)	564	*RAS (KRAS/NRAS)*				OS	87,2%	NA	NA	5-FU with OXA and/or IRI with BEVA or anti-EGFR
Kawaguchi et al. [34]	2019	USA	507				*SMAD4*	OS, RFS	89,7%	NA	NA	NA
Lang et al. [37]	2019	Germany	822	*KRAS*	*BRAF*	*PIK3CA*	*SMAD3, SMAD2, SMAD4*	OS	51,8%	NA	NA	NA
Liu et al. [8]	2019	China	564	*RAS (KRAS/NRAS)*				RFS	100,0%	NA	NA	5-FU with OXA/IRI with or without anti-EGFR or anti-VEGF
Margonis et al. [41]	2019	USA and Europe	1099	*KRAS*	*BRAF*			OS	70,0%	62,6%	NA	NA
O’Connor et al. [48]	2019	Argentina	662	*KRAS*				OS, RFS	28,3%	45,2%	NA	5-FU with OXA/IRI with or without anti-EGFR or anti-VEGF
Ruzzenente et al. [50]	2019	USA and Italy	784	*RAS (KRAS/NRAS)*	*BRAF*			OS	100,0%	48,9%	NA	5-FU with OXA/IRI with or without anti-EGFR or anti-VEGF
Lin et al. [39]	2018	China	139	*KRAS*	*BRAF*			OS, RFS	23,0%	100,0%	23,0%	NA
Margonis et al. [42]	2018	USA and Europe	849	*KRAS*	*BRAF*			OS, RFS	67,3%	50,7%	NA	NA
Mizuno et al. [57]	2018	USA	515	*RAS (KRAS/NRAS)*			*SMAD4*	OS	91,3%	73,0%	NA	5-FU with OXA/IRI with or without anti-EGFR or anti-VEGF
Amikura et al. [22]	2017	Japan	342	*RAS (KRAS/NRAS)*				OS	NA	NA	NA	NA
Serenari et al. [54]	2017	Argentina	26	*KRAS*				OS	96,1%	69,2%	NA	NA
Wang et al. [64]	2017	China	300	*KRAS*				OS	100,0%	100,0%	100,0%	NA
Margonis et al. [46]	2016a	USA (Baltimore)	430	*KRAS*				OS	60,0%	67,8%	43,9%	5-FU with or without OXA/IRI with or without anti-EGFR or anti-VEGF
Margonis et al. [44]	2016b	USA (Baltimore)	512	*KRAS*				RFS	59,3%	69,4%	NA	NA
Brudvik et al. [26]	2016	USA	633	*RAS (KRAS/NRAS)*				OS	86,1%	NA	NA	5-FU with OXA/IRI with BEVA
Sasaki et al. [52]	2016	USA	485	*KRAS*				OS	79,0%	NA	NA	5-FU with OXA/IRI with anti-VEGF or anti-EGFR
Shindoh et al. [55]	2016	Japan	163	*KRAS*				OS, RFS	49,3%	62,2%	NA	NA
Margonis et al. [43]	2015	USA (Baltimore)	334	*KRAS*				OS, RFS	67.7%	62,9%	NA	NA
Zimmitti et al. [60]	2015	USA	184	*RAS (KRAS/NRAS)*				OS	100,0%	NA	NA	5-FU with OXA/IRI with anti-VEGF
Schirripa et al. [53]	2015	Italy	309	*RAS (KRAS/NRAS)*	*BRAF*			OS, RFS	50,0%	21,0%	36,0%	5-FU with OXA/IRI with anti-VEGF or anti-EGFR
Kemeny et al. [36]	2014	USA (New York)	169	*KRAS*				OS, RFS	84,0%	100,0%	NA	5-FU with or without OXA/IRI with HAI
Lin et al. [38]	2014	China	154	*KRAS*	*BRAF*			OS, RFS	24,7%	100,0%	24,7%	NA
Shoji et al. [61]	2014	Japan	108	*KRAS*	*BRAF*	*PIK3CA*		RFS	NA	NA	NA	NA
Isella et al. [30]	2013	Italy	64	*KRAS*		*PIK3CA*		RFS	56,3%	67,2%	20,3%	5-FU with or without OXA/IRI with or without anti-VEGF
Karagkounis et al. [20]	2013	USA (Maryland)	202	*KRAS*				OS, RFS	81,0%	65,0%	NA	NA
Vauthey et al. [59]	2013	USA	193	*RAS (KRAS/NRAS)*		*PIK3CA*		OS, RFS	100,0%	100,0%	100,0%	5-FU with OXA/IRI and anti-VEGF
Stremitzer et al. [56]	2012	Austria	60	*KRAS*				OS	100,0%	100,0%	100,0%	5-FU with OXA and anti-VEGF
Teng et al. [58]	2012	Japan	292	*KRAS*	*BRAF*			OS	22,6%	83,9%	11,3%	5-FU with or without OXA/IRI with or without anti-EGFR
Petrowsky et al. [49]	2001	Germany	41	*KRAS*				OS	NA	NA	NA	5-FU

Note: Abbreviations: BEVA, bevacizumab; CT, chemotherapy; EGFR, epidermal growth factor receptor; 5-FU, 5-fluorouracil; HAI, hepatic arterial infusion; IRI, irinotecan; NA, not available; OS, overall survival; OXA, oxaliplatin, RFS, recurrence-free survival; VEGF, vascular endothelial growth factor.

### Effect of RAS mutation on OS and RFS

Out of 47 studies eligible for the meta-analysis, 43 reported on the association between *RAS* mutation status and patients’ OS and RFS and included a total of 24,121 patients, of whom 8,258 had *RAS* mutations. The pooled *KRAS* mutation rate was 34.2%, which is consistent with previous reports in the literature of resected CRC LM. Specifically, 36 studies reported the association with OS and 20 studies with RFS.

The meta-analysis of OS included 36 studies with 15,766 patients, of whom 5,361 had RAS mutations.

**Figure 2:**
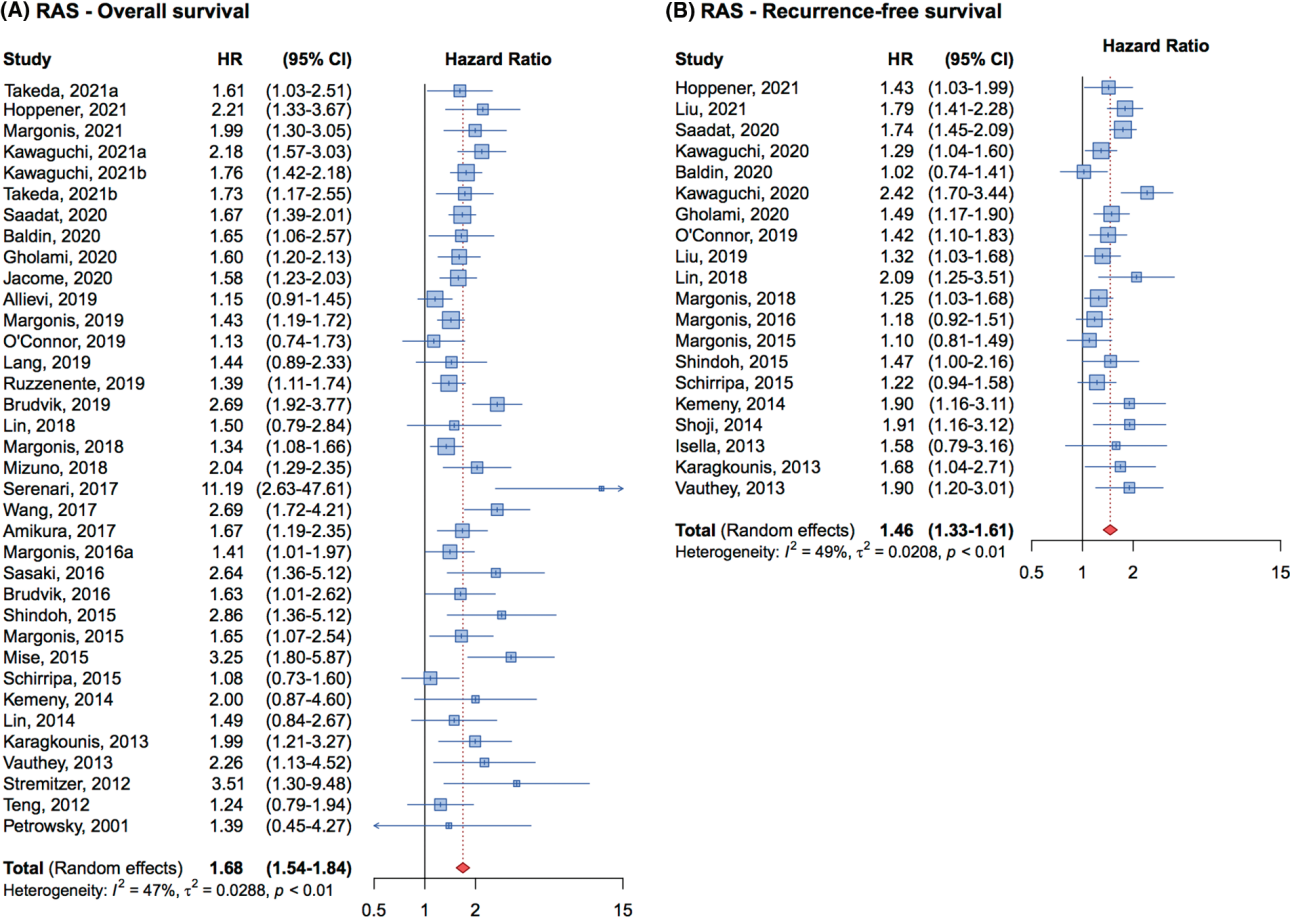
Forrest plot of association between RAS mutation status and overall survival (OS) (A) and recurrence free survival (RFS) (B).

The cumulative HR for OS was 1.68 (95% CI, 1.54–1.84) (Fig. 2A), indicating that *RAS* mutation is an independent prognostic factor associated with poorer OS in patients with metastatic CRC undergoing liver resection.

Regarding RFS, 20 studies involving 8,355 patients were analyzed, with 2,897 patients having RAS mutations. The cumulative HR was 1.46 (95% CI, 1.33–1.61) (Fig. 2B) demonstrating that *RAS* mutations are associated with a higher risk of relapse and a negative impact on prognosis compared to patients with wild-type genes.

An analysis of the forest plot illustrating the effect of *RAS* mutations on RFS in Fig. 2B reveals a notable increase in result homogeneity across different studies beginning in 2019. This uniformity is likely attributable to standardized methodologies in patient enrollment and evaluation across studies, enhancing the reliability of meta-analytic findings.

**Figure 3:**
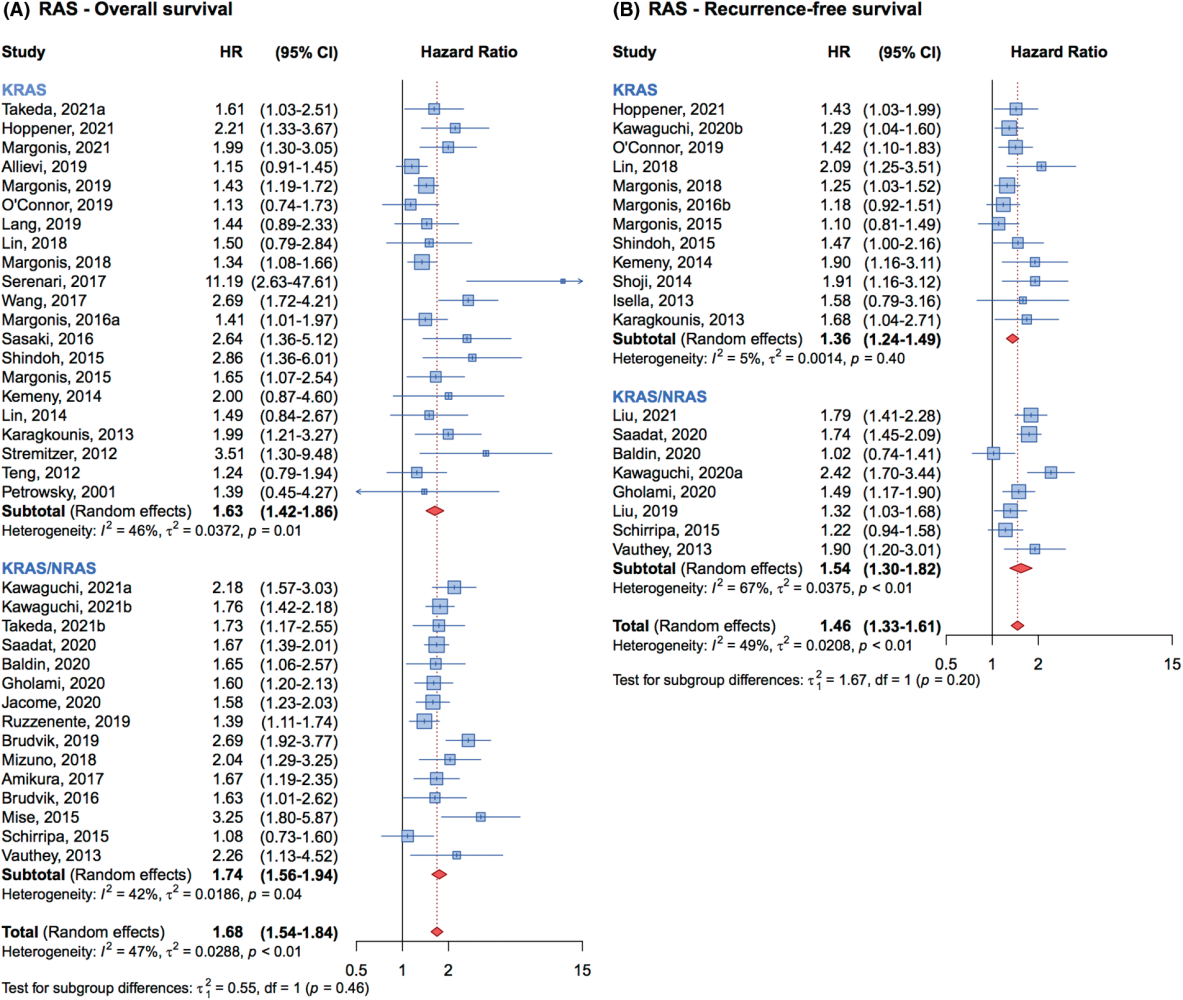
Forrest plot of association between KRAS mutation status *vs*. all-RAS mutation status and overall survival (OS) (A) and recurrence free survival (RFS) (B).

Of the 36 studies included in the meta-analysis on OS, 21 specifically investigated the association between *KRAS* mutation alone and patient OS. The cumulative HR for these studies was 1.63 (95% CI, 1.42–1.86) (Fig. 3A). In contrast, the remaining 15 studies that examined the association between all-*RAS* mutations and patients’ OS, reported a cumulative HR of 1.74 (95% CI, 1.56–1.94) (Fig. 3A). The pooled analysis indicates that the impact on OS is similar whether studies considered only *KRAS* mutations or all *RAS* mutations.

For RFS, the findings were similarly consistent. Twelve studies that focused on KRAS mutations alone reported a cumulative HR of 1.36 (95% CI, 1.24–1.49), while eight studies that evaluated the association between all-RAS mutations and RFS presented a cumulative HR of 1.54 (95% CI, 1.30–1.82) (Fig. 3B). This comparison suggests that the prognostic impact of *KRAS* mutations is comparable to that of all-*RAS* mutations on RFS.

### Effect of BRAF mutation on OS and RFS

Among the 47 studies included in our meta-analysis, 13 reported on OS after CRC LM resection, stratified by *BRAF* mutation status, while 8 studies reported on RFS. The studies included data on 3- or 5-years OS or both, and RFS; 3- or 5-years DFS data were also considered as part of RFS. A total of 8,969 patients were analyzed for *BRAF*-related OS and RFS outcomes, with 433 patients harboring BRAF mutations. The pooled *BRAF* mutation rate was 4.83%, consistent with previous literature on resected CRC LM.

**Figure 4:**
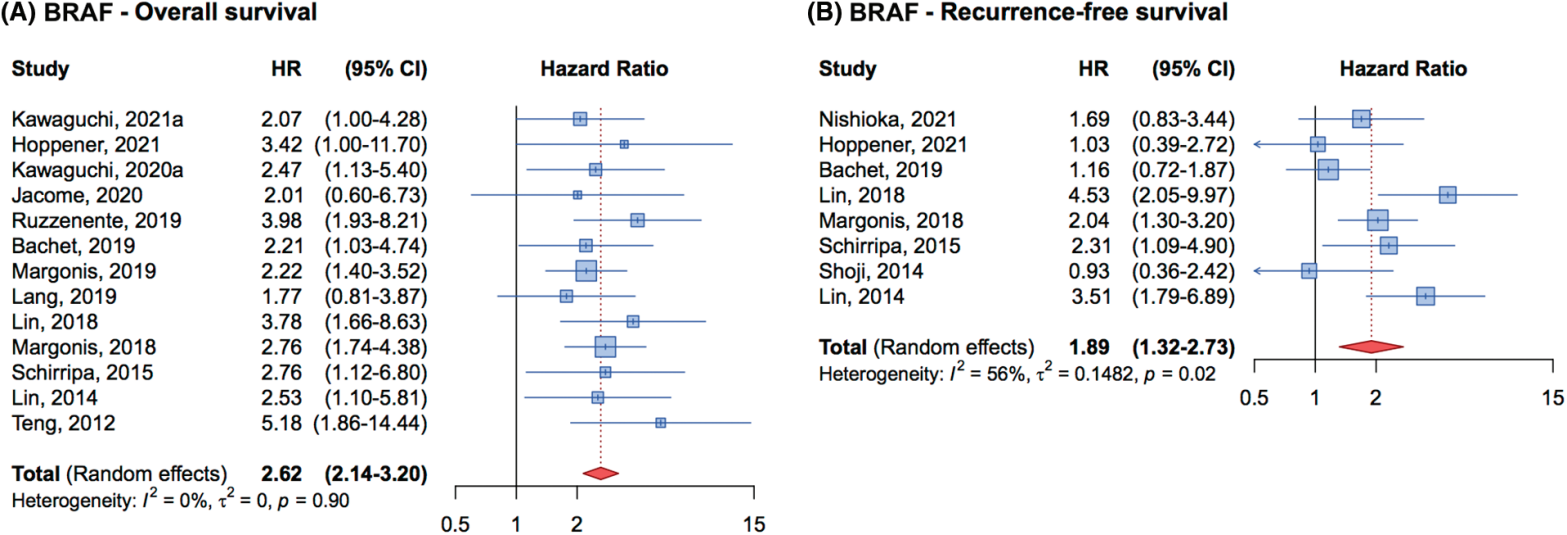
Forrest plot of association between BRAF mutation status and overall survival (OS) (A) and recurrence free survival (RFS) (B).

For the OS, the analysis of 13 studies included 5,831 patients, 270 of whom had *BRAF* mutations. The cumulative HR was 2.62 (95% CI, 2.14–3.20) (Fig. 4A), indicating that the *BRAF* mutation is an independent prognostic factor negatively associated with OS in metastatic CRC patients undergoing complete liver resection.

In terms of RFS, 8 studies with 3,138 patients were analyzed, 163 of whom had BRAF mutations. The cumulative HR was 1.89 (95% CI, 1.32–2.73) (Fig. 4B) demonstrating that *BRAF* mutations are associated with a worse prognosis and a higher risk of relapse compared to wild-type patients.

**Figure 5:**
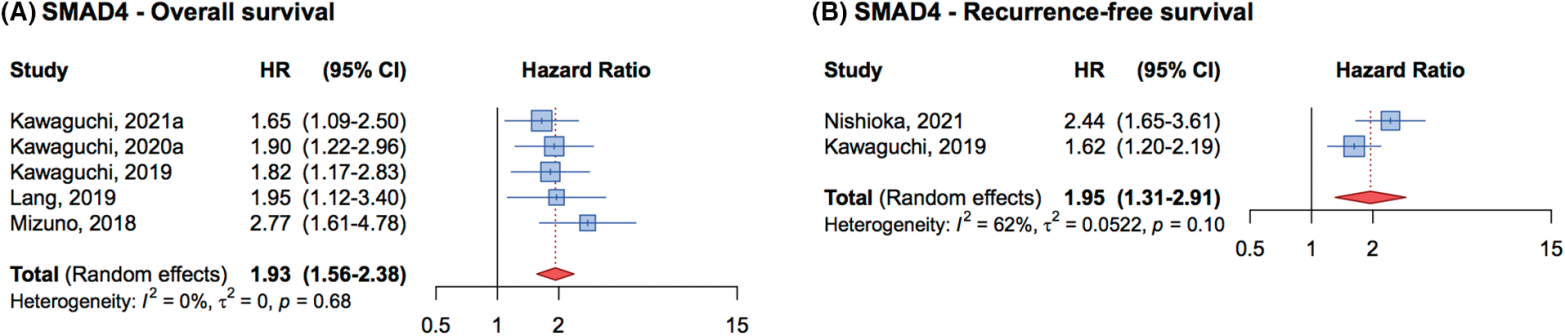
Forrest plot of association between SMAD4 mutation status and overall survival (OS) (A) and recurrence free survival (RFS) (B).

Only 6 studies provided data on OS and/or RFS for CRC LM patients with *SMAD4* mutations. These studies, which focused on *SMAD4* 3-year OS or RFS or DFS, included a total of 3,020 patients in the analysis for both *SMAD4*-related OS and RFS, with 347 harboring a *SMAD4* mutation. Of these, 222 patients were analyzed for OS, and 125 for RFS. The cumulative HR for OS was 1.93 (95% CI, 1.56–2.38) (Fig. 5A), and for RFS was 1.95 (95% CI, 1.31–2.91) (Fig. 5B), confirming *SMAD4* mutations as a detrimental prognostic factor in CRC patients undergoing liver metastasectomy.

### Effect of PIK3CA mutation on OS and RFS

For *PIK3CA* mutations, only 3 studies were deemed eligible for the evaluation of OS and RFS: two addressing RFS and one addressing OS. No significant association with either OS or RFS was found.

### Effect of RAS, BRAF, SMAD4 mutation on OS and RFS according to subgroup of eligibility

No significant differences were observed in the analyses of OS and RFS for studies evaluating *RAS*, *BRAF*, and *SMAD4* mutations, even when data were subdivided according to the subgroup of eligibility (Suppl. Figs. 1–3).

### Other exploratory biomarkers in CRC patients treated with liver metastasectomy: MSI/MMR

Microsatellites, repeated sequences of 1 to 6 base pairs in length composed mostly of non-coding DNA [66,67], are primarily located at chromosome ends and contribute to the individual genetic fingerprint [68].

Microsatellite instability (MSI) results from accumulated errors at microsatellite sites due to a deficient DNA mismatch repair (dMMR) system. This deficiency may arise sporadically, such as through CpG island methylator phenotype (CIMP) due to oxidative stress in *MLH1* promoter hypermethylation, or through inherited conditions like Lynch syndrome or Muir-Torre syndrome [66,69–73]. The MMR system, involving four main genes and their encoded proteins, plays a critical role in DNA replication accuracy by correcting erroneous insertions, deletions, or mis-incorporated bases, thus preventing mismatches between DNA strands [71–74]. Abnormal MMR function leads to frameshift mutations, contributing to a high tumor mutational burden (TMB) and an increase in tumor infiltrating lymphocytes (TILs) [71,74–76].

These factors are pivotal for the effectiveness of immune checkpoint inhibitors in treating unresectable or metastatic tumors with such pathogenesis [77–83]. The meticulous characterization of immune infiltration has become essential for the development of the Immunoscore, a prognostic tool that enhances the TNM classification for high microsatellite instability (MSI-H) CRC [84–87]. Ongoing trials aim to refine survival predictions by introducing new biomarkers [84,88,89].

MSI is associated with various neoplasms, particularly CRC, where it is found in up to 15% of cases, but only 5% in metastatic settings [71,90–93].

Sporadic MSI-H tumors, often identified in older women and associated with the right colon, exhibit distinct features such as poorly differentiated mucinous histology and pronounced lymphocytic infiltration, and a Crohn’s-like host response [66,90,94]. While MSI is a positive prognostic factor in early-stage CRC, its benefit does not extend to metastatic disease, where outcomes are similar to microsatellite stable (MSS) tumors [91,95–99] and dMMR. Recent studies have highlighted the complex role of MSI in predicting intrahepatic recurrences post-liver resection [100], underscoring the need for further research into its prognostic significance alongside other genes. This is in contrast with a previous paper by Haddad et al., which challenged the prognostic significance of MSI, suggesting that the survival of patients with stage IV MSI-positive tumor was not influenced by the surgical resectability of liver metastases [99]. This divergence highlights the complex nature of MSI’s role in CRC and underscores the necessity for more in-depth analysis. Specifically, the relationship between MSI and other commonly examined genes warrants further investigation to clarify its prognostic implications in the context of CRC liver metastases [91,101–103].

### Liver transplantation

The prognostic and predictive value of somatic genetic mutations is under investigation for selecting CRC patients with liver-only metastases for liver transplantation as a curative strategy. Despite the survival benefits observed with surgical treatment, only a minority qualify for resection, and recurrence remains a significant concern. The concept of completely removing the affected liver and performing transplantation emerged in the early eighties but faced challenges due to the high perioperative mortality rate and a significant disease recurrence rate within the first year. However, advancements in the surgical management of CRC, alongside the introduction of fluoropyrimidines-based combination chemotherapy with irinotecan and oxaliplatin (FOLFOX and FOLFIRI achieving response rates around 50%, and the triplet regimen FOLFOXIRI reaching up to 60%) as well as the advent of targeted therapies, have reopened the consideration of liver transplantation as a viable treatment option for patients with metastatic CRC [104].

A landmark in this evolving field was the Secondary Cancer (SECA) I trial, reported in 2013. This trial presented significant survival benefits in a small cohort of stage IV CRC patients with liver-only metastases, documenting 1-, 3-, and 5-year OS rates 95%, 68%, and 60%, respectively, for patients with unresectable liver-only metastases undergoing liver transplantation. The promising results have catalyzed a series of prospective studies aimed at evaluating the feasibility and clinical impact of liver transplantation in patients with surgically inoperable LM from CRC [105].

Particular emphasis has been placed on the molecular criteria for selecting patients for these studies, with the goal of excluding those whose tumors possess characteristics indicating a poorer prognosis. As a result, most ongoing trials require patients to have tumors that are wild-type for both *RAS* and *BRAF* genes.

The results of the Italian COLT trial were recently published [106]. It demonstrated that liver transplantation in metastatic CRC, coupled with improved patients selection strategies including tumor molecular characterization for RAS/RAF can give patients a 5-year OS similar to other indications for liver transplantation and a better outcome than those undergoing chemotherapy alone [106].

## Discussion

Significant advancements in survival rates for patients with metastatic CRC through the introduction of new chemotherapy combination regimens and targeted agents such as cetuximab and bevacizumab, coupled with an enhanced molecular understanding of cancer biology. Nonetheless, surgical intervention remains the sole potentially curative option for patients with oligometastases confined to a single organ, such as the liver. Surgical and oncological criteria are employed to properly select patients for whom the liver metastasectomy is feasible and who are likely to derive a sustained benefit from the surgical procedure. The evolving knowledge of CRC’s molecular biology and liver metastases has unveiled crucial somatic alterations in specific genes, specifically affecting patients’ prognosis and their tumors’ response to pharmacological treatments. While these findings are integral to therapy selection, their role in determining suitability for liver metastasectomy continues to be explored.

Our metanalysis reaffirmed that mutations in *RAS*, particularly *BRAF*, serve as independent prognostic factors, diminishing OS and RFS in patients with CRCLM who undergo resection.

These findings align with previous meta-analyses that advocate for incorporating molecular profiling into patient selection for liver surgery [19,107–109].

In evaluating *RAS* mutations, we also considered the impact of testing solely for *KRAS* mutations versus. assessing the entire *RAS* gene family. Our analysis indicated that patients with any *RAS* mutation exhibited similar OS and RFS outcomes to those with only *KRAS* mutations.

To our knowledge, this meta-analysis is the most up-to-date and comprehensive, extending beyond *RAS* and *BRAF* mutations to include additional biomarkers. We also examined the role of *SMAD4* and *PIK3CA*, two significant factors in CRC carcinogenesis. Although only a few studies addressed this topic, three recent ones highlighted the adverse impact of *SMAD4* mutations on OS following liver metastasectomy. Conversely, data on the effect of *SMAD4* on RFS were unavailable. Regarding *PIK3CA* mutations, only two studies were found, yielding inconclusive results on their prognostic significance after liver metastasectomy.

Our meta-analysis underscores no significant differences in OS and RFS across subgroups of eligibility, supporting our overall findings. However, several limitations are noted: 1) the pooling of results from studies assessing patient outcomes as either DFS or RFS, despite their statistical differences; 2) the inclusion of patients with minimal residual disease, where positive resection margins significantly impact prognosis; 3) potential confounding factors not considered, such as variability in resectability definitions, chemotherapy use, and other variables in multivariable analyses; 4) a lack of specificity in some studies regarding the *BRAF* mutations examined; 5) an unassessed potential interaction between biological markers and pre- and post-resection pharmacological treatments due to treatment heterogeneity. For patients ineligible for primary hepatic metastasectomy due to extensive liver involvement, liver transplantation is being considered in ongoing clinical trials. Here, the tumor’s underlying biology, especially *RAF/RAS* status, is crucial for patient selection in experimental liver transplantation protocols. Additionally, other tumor molecular characteristics, such as MMR proficiency status, which has already shown predictive and prognostic value in CRC, could be evaluated as prognostic markers for patients undergoing liver resection. These could eventually serve as additional criteria for patient selection, potentially enhancing the clinical management of metastatic CRC patients.

## Conclusions

Mutational status of *BRAF*, *RAS*, and *SMAD4* in tumor tissue from mCRC patients candidate for liver metastasectomy should be considered to select those patients with a higher chance of benefiting from the surgical treatment. This could spare useless procedures and complications in patients with high disease recurrence chances. Tumor mutational status could also inform clinicians of the potential benefit of a liver transplantation procedure. The effect of *PIK3CA* mutations on the outcome of hepatic metastasectomy is still controversial and should be better investigated in future studies.

## Supplementary Materials

Figure S1 RAS overall survival and recurrence - free survival subdivided for eligibility.

Figure S2 BRAF overall survival and recurrence - free survival subdivided for eligibility.

Figure S3 SMAD4 overall survival and recurrence - free survival subdivided for eligibility.

## Data Availability

Not applicable.

## Supplementary Materials

The supplementary material is available online at https://doi.org/10.32604/or.2024.049181.

## References

[1] Siegel, R. L., Wagle, N. S., Cercek, A., Smith, R. A., Jemal, A. (2023). Colorectal cancer statistics, 2023. CA: A Cancer Journal for Clinicians*,* 73 *(*3*),* 233–254. 10.3322/caac.21772; 36856579

[2] Sargent, D., Sobrero, A., Grothey, A., O’Connell, M. J., Buyse, M. et al. (2009). Evidence for cure by adjuvant therapy in colon cancer: Observations based on individual patient data from 20,898 patients on 18 randomized trials. Journal of Clinical Oncology*,* 27 *(*6*),* 872–877. 10.1200/JCO.2008.19.5362; 19124803 PMC2738431

[3] Modest, D. P., Pant, S., Sartore-Bianchi, A. (2019). Treatment sequencing in metastatic colorectal cancer. European Journal of Cancer*,* 109*,* 70–83. 10.1016/j.ejca.2018.12.019; 30690295

[4] Rebersek, M. (2020). Consensus molecular subtypes (CMS) in metastatic colorectal cancer—Personalized medicine decision. Radiology and Oncology*,* 54 *(*3*),* 272–277. 10.2478/raon-2020-0031; 32463385 PMC7409603

[5] Morris, V. K., Kennedy, E. B., Baxter, N. N., Benson, A. B., Cercek, A. et al. (2023). Treatment of metastatic colorectal cancer: ASCO guideline. Journal of Clinical Oncology*,* 41 *(*3*),* 678–700. 10.1200/JCO.22.01690; 36252154 PMC10506310

[6] Adam, R., Wicherts, D. A., de Haas, R. J., Aloia, T., Lévi, F. et al. (2008). Complete pathologic response after preoperative chemotherapy for colorectal liver metastases: Myth or reality? Journal of Clinical Oncology*,* 26 *(*10*),* 1635–1641. 10.1200/JCO.2007.13.7471; 18375892

[7] Tsilimigras, D. I., Ntanasis-Stathopoulos, I., Bagante, F., Moris, D., Cloyd, J. et al. (2018). Clinical significance and prognostic relevance of KRAS, BRAF, PI3K and TP53 genetic mutation analysis for resectable and unresectable colorectal liver metastases: A systematic review of the current evidence. Surgical Oncology*,* 27 *(*2*),* 280–288. 10.1016/j.suronc.2018.05.012; 29937183

[8] Liu, W., Wang, K., Han, Y., Liang, J. Y., Li, Y. H. et al. (2019). Nomogram predicted disease free survival for colorectal liver metastasis patients with preoperative chemotherapy followed by hepatic resection. European Journal of Surgical Oncology*,* 45 *(*11*),* 2070–2077. 10.1016/j.ejso.2019.06.033; 31279595

[9] Jones, R. P., Brudvik, K. W., Franklin, J. M., Poston, G. J. (2017). Precision surgery for colorectal liver metastases: Opportunities and challenges of omics-based decision making. European Journal of Surgical Oncology*,* 43 *(*5*),* 875–883. 10.1016/j.ejso.2017.02.014; 28302330

[10] Peeters, M., Kafatos, G., Taylor, A., Gastanaga, V. M., Oliner, K. S. et al. (2015). Prevalence of RAS mutations and individual variation patterns among patients with metastatic colorectal cancer: A pooled analysis of randomised controlled trials. European Journal of Cancer*,* 51 *(*13*),* 1704–1713. 10.1016/j.ejca.2015.05.017; 26049686

[11] Ucar, G., Ergun, Y., Aktürk Esen, S., Acikgoz, Y., Dirikoc, M. et al. (2020). Prognostic and predictive value of KRAS mutation number in metastatic colorectal cancer. Medicine*,* 99 *(*39*),* e22407. 10.1097/MD.0000000000022407; 32991469 PMC7523797

[12] Cohen, R., Cervera, P., Svrcek, M., Pellat, A., Dreyer, C. et al. (2017). BRAF-mutated colorectal cancer: What is the optimal strategy for treatment? Current Treatment Options in Oncology*,* 18 *(*2*),* 9. 10.1007/s11864-017-0453-5; 28214977

[13] Chen, D., Huang, J. F., Liu, K., Zhang, L. Q., Yang, Z. et al. (2014). BRAFV600E mutation and its association with clinicopathological features of colorectal cancer: A systematic review and meta-analysis. PLoS One*,* 9 *(*3*),* e90607. 10.1371/journal.pone.0090607; 24594804 PMC3940924

[14] Pietrantonio, F., Cremolini, C., Petrelli, F., Di Bartolomeo, M., Loupakis, F. et al. (2015). First-line anti-EGFR monoclonal antibodies in panRAS wild-type metastatic colorectal cancer: A systematic review and meta-analysis. Critical Reviews in Oncology/Hematology*,* 96 *(*1*),* 156–166. 10.1016/j.critrevonc.2015.05.016; 26088456

[15] Zaniboni, A., Formica, V. (2016). The best. First. Anti-EGFR before anti-VEGF, in the first-line treatment of RAS wild-type metastatic colorectal cancer: From bench to bedside. Cancer Chemotherapy and Pharmacology*,* 78 *(*2*),* 233–244. 10.1007/s00280-016-3032-8; 27091467

[16] van Brummelen, E. M. J., de Boer, A., Beijnen, J. H., Schellens, J. H. M. (2017). BRAF mutations as predictive biomarker for response to anti-EGFR monoclonal antibodies. The Oncologist*,* 22 *(*7*),* 864–872. 10.1634/theoncologist.2017-0031; 28576857 PMC5507642

[17] Cremolini, C., Loupakis, F., Antoniotti, C., Lupi, C., Sensi, E. et al. (2015). FOLFOXIRI plus bevacizumab versus FOLFIRI plus bevacizumab as first-line treatment of patients with metastatic colorectal cancer: Updated overall survival and molecular subgroup analyses of the open-label, phase 3 TRIBE study. The Lancet Oncology*,* 16 *(*13*),* 1306–1315. 10.1016/S1470-2045(15)00122-9; 26338525

[18] Loupakis, F., Intini, R., Cremolini, C., Orlandi, A., Sartore-Bianchi, A. et al. (2019). A validated prognostic classifier for ^V600E^*BRAF*-mutated metastatic colorectal cancer: The «BRAF BeCool» study. European Journal of Cancer*,* 118*,* 121–130. 10.1016/j.ejca.2019.06.008; 31330487

[19] Tosi, F., Magni, E., Amatu, A., Mauri, G., Bencardino, K. et al. (2017). Effect of KRAS and BRAF mutations on survival of metastatic colorectal cancer after liver resection: A systematic review and meta-analysis. Clinical Colorectal Cancer*,* 16 *(*3*),* e153–e163. 10.1016/j.clcc.2017.01.004; 28216246

[20] Karagkounis, G., Torbenson, M. S., Daniel, H. D., Azad, N. S., Diaz, L. A. et al. (2013). Incidence and prognostic impact of KRAS and BRAF mutation in patients undergoing liver surgery for colorectal metastases. Cancer*,* 119 *(*23*),* 4137–4144. 10.1002/cncr.28347; 24104864 PMC3967132

[21] Allievi, N., Goffredo, P., Utria, A. F., Pisano, M., Poiasina, E. et al. (2019). The association of KRAS mutation with primary tumor location and survival in patients undergoing resection of colorectal cancers and synchronous liver metastases. Chinese Clinical Oncology*,* 8 *(*5*),* 46. 10.21037/cco.2019.08.10; 31500429

[22] Amikura, K., Akagi, K., Ogura, T., Takahashi, A., Sakamoto, H. (2018). The RAS mutation status predicts survival in patients undergoing hepatic resection for colorectal liver metastases: The results from a genetic analysis of all-RAS. Journal of Surgical Oncology*,* 117 *(*4*),* 745–755. 10.1002/jso.24910; 29194647

[23] Bachet, J. B., Moreno-Lopez, N., Vigano, L., Marchese, U., Gelli, M. et al. (2019). BRAF mutation is not associated with an increased risk of recurrence in patients undergoing resection of colorectal liver metastases. The British Journal of Surgery*,* 106 *(*9*),* 1237–1247. 10.1002/bjs.11180; 31183866

[24] Baldin, P., van den Eynde, M., Mlecnik, B., Bindea, G., Beniuga, G. et al. (2021). Prognostic assessment of resected colorectal liver metastases integrating pathological features, RAS mutation and immunoscore. The Journal of Pathology: Clinical Research*,* 7 *(*1*),* 27–41. 10.1002/cjp2.178; 32902189 PMC7737782

[25] Brudvik, K. W., Jones, R. P., Giuliante, F., Shindoh, J., Passot, G. et al. (2019). RAS mutation clinical risk score to predict survival after resection of colorectal liver metastases. Annals of Surgery*,* 269 *(*1*),* 120–126. 10.1097/SLA.0000000000002319; 28549012

[26] Brudvik, K. W., Mise, Y., Chung, M. H., Chun, Y. S., Kopetz, S. E. et al. (2016). RAS mutation predicts positive resection margins and narrower resection margins in patients undergoing resection of colorectal liver metastases. Annals of Surgical Oncology*,* 23 *(*8*),* 2635–2643. 10.1245/s10434-016-5187-2; 27016292 PMC5527830

[27] Brunsell, T. H., Sveen, A., Bjørnbeth, B. A., Røsok, B. I., Danielsen, S. A. et al. (2020). High concordance and negative prognostic impact of RAS/BRAF/PIK3CA mutations in multiple resected colorectal liver metastases. Clinical Colorectal Cancer*,* 19 *(*1*),* e26–e47. 10.1016/j.clcc.2019.09.003; 31982351

[28] Gholami, S., Stewart, S., Kemeny, N., Gönen, M., Groot Koerkamp, B. et al. (2021). Impact of primary tumor laterality on adjuvant hepatic artery infusion pump chemotherapy in resected colon cancer liver metastases: Analysis of 487 patients. Annals of Surgical Oncology*,* 28 *(*7*),* 3685–3694. 10.1245/s10434-020-09369-7; 33230748 PMC8385634

[29] Höppener, D. J., Galjart, B., Nierop, P. M. H., Buisman, F. E., van der Stok, E. P., et al. (2021). Histopathological growth patterns and survival after resection of colorectal liver metastasis: An external validation study. JNCI Cancer Spectrum*,* 5 *(*3*),* pkab026. 10.1093/jncics/pkab026; 34056541 PMC8152695

[30] Isella, C., Mellano, A., Galimi, F., Petti, C., Capussotti, L. et al. (2013). MACC1 mRNA levels predict cancer recurrence after resection of colorectal cancer liver metastases. Annals of Surgery*,* 257 *(*6*),* 1089–1095. 10.1097/SLA.0b013e31828f96bc; 23665971

[31] Jácome, A. A., Vreeland, T. J., Johnson, B., Kawaguchi, Y., Wei, S. H. et al. (2021). The prognostic impact of RAS on overall survival following liver resection in early versus late-onset colorectal cancer patients. British Journal of Cancer*,* 124 *(*4*),* 797–804. 10.1038/s41416-020-01169-w; 33208919 PMC7884678

[32] Kawaguchi, Y., Kopetz, S., Kwong, L., Xiao, L., Morris, J. S. et al. (2021). Genomic sequencing and insight into clinical heterogeneity and prognostic pathway genes in patients with metastatic colorectal cancer. Journal of the American College of Surgeons*,* 233 *(*2*),* 272–284.e13. 10.1016/j.jamcollsurg.2021.05.027; 34111531 PMC8666966

[33] Kawaguchi, Y., Kopetz, S., Tran Cao, H. S., Panettieri, E., de Bellis, M. et al. (2021). Contour prognostic model for predicting survival after resection of colorectal liver metastases: Development and multicentre validation study using largest diameter and number of metastases with RAS mutation status. The British Journal of Surgery*,* 108 *(*8*),* 968–975. 10.1093/bjs/znab086; 33829254 PMC8378514

[34] Kawaguchi, Y., Kopetz, S., Newhook, T. E., de Bellis, M., Chun, Y. S. et al. (2019). Mutation status of RAS, TP53, and SMAD4 is superior to mutation status of RAS alone for predicting prognosis after resection of colorectal liver metastases. Clinical Cancer Research*,* 25 *(*19*),* 5843–5851. 10.1158/1078-0432.CCR-19-0863; 31221662 PMC6774854

[35] Kawaguchi, Y., Kopetz, S., Lillemoe, H. A., Hwang, H., Wang, X. et al. (2020). A new surveillance algorithm after resection of colorectal liver metastases based on changes in recurrence risk and RAS mutation status. Journal of the National Comprehensive Cancer Network*,* 18 *(*11*),* 1500–1508. 10.6004/jnccn.2020.7596; 33152698 PMC10547101

[36] Kemeny, N. E., Chou, J. F., Capanu, M., Gewirtz, A. N., Cercek, A. et al. (2014). KRAS mutation influences recurrence patterns in patients undergoing hepatic resection of colorectal metastases. Cancer*,* 120 *(*24*),* 3965–3971. 10.1002/cncr.28954; 25155157 PMC4574496

[37] Lang, H., Baumgart, J., Heinrich, S., Tripke, V., Passalaqua, M. et al. (2019). Extended molecular profiling improves stratification and prediction of survival after resection of colorectal liver metastases. Annals of Surgery*,* 270 *(*5*),* 799–805. 10.1097/SLA.0000000000003527; 31634180

[38] Lin, Q., Ye, Q., Zhu, D., Wei, Y., Ren, L. et al. (2014). Determinants of long-term outcome in patients undergoing simultaneous resection of synchronous colorectal liver metastases. PLoS One*,* 9 *(*8*),* e105747. 10.1371/journal.pone.0105747; 25162714 PMC4146540

[39] Lin, Q., Jian, M., Niu, Z. C., Xu, P. P., Zheng, P. et al. (2018). Prognostic impact of KRAS and BRAF mutations in patients who underwent simultaneous resection for initially resectable colorectal liver metastases. International Journal of Clinical and Experimental Pathology*,* 11 *(*12*),* 5981–5991; 31949686 PMC6963078

[40] Liu, W., Zhang, W., Xu, Y., Li, Y. H., Xing, B. C. (2021). A prognostic scoring system to predict survival outcome of resectable colorectal liver metastases in this modern era. Annals of Surgical Oncology*,* 28 *(*12*),* 7709–7718. 10.1245/s10434-021-10143-6; 34023948

[41] Margonis, G. A., Buettner, S., Andreatos, N., Wagner, D., Sasaki, K. et al. (2019). Prognostic factors change over time after hepatectomy for colorectal liver metastases: A multi-institutional, international analysis of 1099 patients. Annals of Surgery*,* 269 *(*6*),* 1129–1137. 10.1097/SLA.0000000000002664; 31082912

[42] Margonis, G. A., Buettner, S., Andreatos, N., Kim, Y., Wagner, D. et al. (2018). Association of BRAF mutations with survival and recurrence in surgically treated patients with metastatic colorectal liver cancer. JAMA Surgery*,* 153 *(*7*),* e180996. 10.1001/jamasurg.2018.0996; 29799910 PMC6137519

[43] Margonis, G. A., Spolverato, G., Kim, Y., Karagkounis, G., Choti, M. A. et al. (2015). Effect of KRAS mutation on long-term outcomes of patients undergoing hepatic resection for colorectal liver metastases. Annals of Surgical Oncology*,* 22 *(*13*),* 4158–4165. 10.1245/s10434-015-4587-z; 26077912

[44] Margonis, G. A., Kim, Y., Sasaki, K., Samaha, M., Amini, N. et al. (2016). Codon 13 KRAS mutation predicts patterns of recurrence in patients undergoing hepatectomy for colorectal liver metastases. Cancer*,* 122 *(*17*),* 2698–2707. 10.1002/cncr.30085; 27244540

[45] Margonis, G. A., Amini, N., Buettner, S., Kim, Y., Wang, J. et al. (2021). The prognostic impact of primary tumor site differs according to the KRAS mutational status: A study by the international genetic consortium for colorectal liver metastasis. Annals of Surgery*,* 273 *(*6*),* 1165–1172. 10.1097/SLA.0000000000003504; 31389831

[46] Margonis, G. A., Kim, Y., Sasaki, K., Samaha, M., Buettner, S. et al. (2016). Activating KRAS mutation is prognostic only among patients who receive preoperative chemotherapy before resection of colorectal liver metastases. Journal of Surgical Oncology*,* 114 *(*3*),* 361–367. 10.1002/jso.24319; 27264476

[47] Nishioka, Y., Paez-Arango, N., Boettcher, F. O., Kawaguchi, Y., Newhook, T. E. et al. (2022). Neither surgical margin status nor somatic mutation predicts local recurrence after R0-intent resection for colorectal liver metastases. Journal of Gastrointestinal Surgery*,* 26 *(*4*),* 791–801. 10.1007/s11605-021-05173-0; 34725784 PMC11875739

[48] O’Connor, J. M., Sanchez Loria, F., Ardiles, V., Grondona, J., Sanchez, P. et al. (2019). Prognostic impact of K-RAS mutational status and primary tumor location in patients undergoing resection for colorectal cancer liver metastases: An update. Future Oncology*,* 15 *(*27*),* 3149–3157. 10.2217/fon-2019-0196; 31426677

[49] Petrowsky, H., Sturm, I., Graubitz, O., Kooby, D. A., Staib-Sebler, E. et al. (2001). Relevance of Ki-67 antigen expression and K-ras mutation in colorectal liver metastases. European Journal of Surgical Oncology*,* 27 *(*1*),* 80–87. 10.1053/ejso.2000.1029; 11237496

[50] Ruzzenente, A., Bagante, F., Ratti, F., Beal, E. W., Alexandrescu, S. et al. (2019). Response to preoperative chemotherapy: Impact of change in total burden score and mutational tumor status on prognosis of patients undergoing resection for colorectal liver metastases. HPB*,* 21 *(*9*),* 1230–1239. 10.1016/j.hpb.2019.01.014; 30792047

[51] Saadat, L. V., Boerner, T., Goldman, D. A., Gonen, M., Frankel, T. L. et al. (2021). Association of RAS mutation location and oncologic outcomes after resection of colorectal liver metastases. Annals of Surgical Oncology*,* 28 *(*2*),* 817–825. 10.1245/s10434-020-08862-3; 32683635 PMC7854850

[52] Sasaki, K., Margonis, G. A., Andreatos, N., Kim, Y., Wilson, A. et al. (2016). Combined resection and RFA in colorectal liver metastases: Stratification of long-term outcomes. The Journal of Surgical Research*,* 206 *(*1*),* 182–189. 10.1016/j.jss.2016.06.098; 27916360

[53] Schirripa, M., Bergamo, F., Cremolini, C., Casagrande, M., Lonardi, S. et al. (2015). BRAF and RAS mutations as prognostic factors in metastatic colorectal cancer patients undergoing liver resection. British Journal of Cancer*,* 112 *(*12*),* 1921–1928. 10.1038/bjc.2015.142; 25942399 PMC4580391

[54] Serenari, M., Alvarez, F. A., Ardiles, V., de Santibañes, M., Pekolj, J. et al. (2018). The ALPPS approach for colorectal liver metastases: Impact of KRAS mutation status in survival. Digestive Surgery*,* 35 *(*4*),* 303–310. 10.1159/000471930; 29032374

[55] Shindoh, J., Nishioka, Y., Yoshioka, R., Sugawara, T., Sakamoto, Y. et al. (2016). KRAS mutation status predicts site-specific recurrence and survival after resection of colorectal liver metastases irrespective of location of the primary lesion. Annals of Surgical Oncology*,* 23 *(*6*),* 1890–1896. 10.1245/s10434-016-5087-5; 26786089

[56] Stremitzer, S., Stift, J., Gruenberger, B., Tamandl, D., Aschacher, T. et al. (2012). KRAS status and outcome of liver resection after neoadjuvant chemotherapy including bevacizumab. The British Journal of Surgery*,* 99 *(*11*),* 1575–1582. 10.1002/bjs.8909; 23027075

[57] Mizuno, T., Cloyd, J. M., Vicente, D., Omichi, K., Chun, Y. S. et al. (2018). SMAD4 gene mutation predicts poor prognosis in patients undergoing resection for colorectal liver metastases. European Journal of Surgical Oncology*,* 44 *(*5*),* 684–692. 10.1016/j.ejso.2018.02.247; 29551247

[58] Teng, H. W., Huang, Y. C., Lin, J. K., Chen, W. S., Lin, T. C. et al. (2012). BRAF mutation is a prognostic biomarker for colorectal liver metastasectomy. Journal of Surgical Oncology*,* 106 *(*2*),* 123–129. 10.1002/jso.23063; 22331825

[59] Vauthey, J. N., Zimmitti, G., Kopetz, S. E., Shindoh, J., Chen, S. S. et al. (2013). RAS mutation status predicts survival and patterns of recurrence in patients undergoing hepatectomy for colorectal liver metastases. Annals of Surgery*,* 258 *(*4*),* 619–627. 10.1097/SLA.0b013e3182a5025a; 24018645 PMC3856211

[60] Zimmitti, G., Shindoh, J., Mise, Y., Kopetz, S., Loyer, E. M. et al. (2015). RAS mutations predict radiologic and pathologic response in patients treated with chemotherapy before resection of colorectal liver metastases. Annals of Surgical Oncology*,* 22 *(*3*),* 834–842. 10.1245/s10434-014-4042-6; 25227306 PMC4318708

[61] Shoji, H., Yamada, Y., Taniguchi, H., Nagashima, K., Okita, N. et al. (2014). Clinical impact of c-MET expression and genetic mutational status in colorectal cancer patients after liver resection. Cancer Science*,* 105 *(*8*),* 1002–1007. 10.1111/cas.12453; 24863535 PMC4317860

[62] Takeda, Y., Mise, Y., Takahashi, Y., Ito, H., Inoue, Y. et al. (2022). Limited prognostic value of KRAS in patients undergoing hepatectomy for colorectal liver metastases. Annals of Surgical Oncology*,* 29 *(*4*),* 2383–2391. 10.1245/s10434-021-11015-9; 34851437

[63] Takeda, Y., Mise, Y., Matsumura, M., Hasegawa, K., Yoshimoto, J. et al. (2021). Accuracy of modern clinical risk score including RAS status changes based on whether patients received perioperative chemotherapy for colorectal liver metastases. World Journal of Surgery*,* 45 *(*7*),* 2176–2184. 10.1007/s00268-021-05976-x; 33880608

[64] Wang, K., Liu, W., Yan, X. L., Li, J., Xing, B. C. (2017). Long-term postoperative survival prediction in patients with colorectal liver metastasis. Oncotarget*,* 8 *(*45*),* 79927–79934. 10.18632/oncotarget.20322; 29108374 PMC5668107

[65] Kawaguchi, Y., Newhook, T. E., Tran Cao, H. S., Tzeng, C. W. D., Chun, Y. S. et al. (2021). Alteration of FBXW7 is associated with worse survival in patients undergoing resection of colorectal liver metastases. Journal of Gastrointestinal Surgery*,* 25 *(*1*),* 186–194. 10.1007/s11605-020-04866-2; 33205306 PMC10095595

[66] Buecher, B., Cacheux, W., Rouleau, E., Dieumegard, B., Mitry, E. et al. (2013). Role of microsatellite instability in the management of colorectal cancers. Digestive and Liver Disease*,* 45 *(*6*),* 441–449. 10.1016/j.dld.2012.10.006; 23195666

[67] Zhang, L. (2008). Immunohistochemistry versus microsatellite instability testing for screening colorectal cancer patients at risk for hereditary nonpolyposis colorectal cancer syndrome. Part II. The utility of microsatellite instability testing. Journal of Molecular Diagnostic*,* 10 *(*4*),* 293–300. 10.2353/jmoldx.2008.080062; 18556767 PMC2438196

[68] Ellegren, H. (2004). Microsatellites: Simple sequences with complex evolution. Nature Reviews Genetics*,* 5 *(*6*),* 435–445. 10.1038/nrg1348; 15153996

[69] Tiwari, A. K., Roy, H. K., Lynch, H. T. (2016). Lynch syndrome in the 21^st^ century: Clinical perspectives. QJM*,* 109 *(*3*),* 151–158. 10.1093/qjmed/hcv137; 26224055

[70] Kocarnik, J. M., Shiovitz, S., Phipps, A. I. (2015). Molecular phenotypes of colorectal cancer and potential clinical applications. Gastroenterology Report*,* 3 *(*4*),* 269–276. 10.1093/gastro/gov046; 26337942 PMC4650976

[71] Boland, C. R., Goel, A. (2010). Microsatellite instability in colorectal cancer. Gastroenterology*,* 138 *(*6*),* 2073–2087. 10.1053/j.gastro.2009.12.064; 20420947 PMC3037515

[72] Weisenberger, D. J., Siegmund, K. D., Campan, M., Young, J., Long, T. I. et al. (2006). CpG island methylator phenotype underlies sporadic microsatellite instability and is tightly associated with BRAF mutation in colorectal cancer. Nature Genetics*,* 38 *(*7*),* 787–793. 10.1038/ng1834; 16804544

[73] Ward, R., Meagher, A., Tomlinson, I., O’Connor, T., Norrie, M. et al. (2001). Microsatellite instability and the clinicopathological features of sporadic colorectal cancer. Gut*,* 48 *(*6*),* 821–829. 10.1136/gut.48.6.821; 11358903 PMC1728324

[74] Baretti, M., Le, D. T. (2018). DNA mismatch repair in cancer. Pharmacology & Therapeutics*,* 189*,* 45–62. 10.1016/j.pharmthera.2018.04.004; 29669262

[75] Chan, T. A., Yarchoan, M., Jaffee, E., Swanton, C., Quezada, S. A. et al. (2019). Development of tumor mutation burden as an immunotherapy biomarker: Utility for the oncology clinic. Annals of Oncology*,* 30 *(*1*),* 44–56. 10.1093/annonc/mdy495; 30395155 PMC6336005

[76] Armaghany, T., Wilson, J. D., Chu, Q., Mills, G. (2012). Genetic alterations in colorectal cancer. Gastrointestinal Cancer Research*,* 5 *(*1*),* 19–27; 22574233 PMC3348713

[77] Schrock, A. B., Ouyang, C., Sandhu, J., Sokol, E., Jin, D. et al. (2019). Tumor mutational burden is predictive of response to immune checkpoint inhibitors in MSI-high metastatic colorectal cancer. Annals of Oncology*,* 30 *(*7*),* 1096–1103. 10.1093/annonc/mdz134; 31038663

[78] Ciardiello, D., Vitiello, P. P., Cardone, C., Martini, G., Troiani, T. et al. (2019). Immunotherapy of colorectal cancer: Challenges for therapeutic efficacy. Cancer Treatment Reviews*,* 76*,* 22–32. 10.1016/j.ctrv.2019.04.003; 31079031

[79] Maleki Vareki, S. (2018). High and low mutational burden tumors versus immunologically hot and cold tumors and response to immune checkpoint inhibitors. Journal of Immunotherapy of Cancer*,* 6 *(*1*),* 157. 10.1186/s40425-018-0479-7; 30587233 PMC6307306

[80] Overman, M. J., McDermott, R., Leach, J. L., Lonardi, S., Lenz, H. J. et al. (2017). Nivolumab in patients with metastatic DNA mismatch repair-deficient or microsatellite instability-high colorectal cancer (CheckMate 142): An open-label, multicentre, phase 2 study. The Lancet Oncology*,* 18 *(*9*),* 1182–1191. 10.1016/S1470-2045(17)30422-9; 28734759 PMC6207072

[81] Overman, M. J., Lonardi, S., Wong, K. Y. M., Lenz, H. J., Gelsomino, F. et al. (2018). Durable clinical benefit with nivolumab plus ipilimumab in DNA mismatch repair-deficient/microsatellite instability-high metastatic colorectal cancer. Journal of Clinical Oncology*,* 36 *(*8*),* 773–779. 10.1200/JCO.2017.76.9901; 29355075

[82] Lipson, E. J., Sharfman, W. H., Drake, C. G., Wollner, I., Taube, J. M. et al. (2013). Durable cancer regression off-treatment and effective reinduction therapy with an anti-PD-1 antibody. Clinical Cancer Research*,* 19 *(*2*),* 462–468. 10.1158/1078-0432.CCR-12-2625; 23169436 PMC3548952

[83] Brahmer, J. R., Drake, C. G., Wollner, I., Powderly, J. D., Picus, J. et al. (2010). Phase I study of single-agent anti-programmed death-1 (MDX-1106) in refractory solid tumors: Safety, clinical activity, pharmacodynamics, and immunologic correlates. Journal of Clinical Oncology*,* 28 *(*19*),* 3167–3175. 10.1200/JCO.2009.26.7609; 20516446 PMC4834717

[84] Yomoda, T., Sudo, T., Kawahara, A., Shigaki, T., Shimomura, S. et al. (2019). The immunoscore is a superior prognostic tool in stages II and III colorectal cancer and is significantly correlated with programmed death-ligand 1 (PD-L1) expression on tumor-infiltrating mononuclear cells. Annals of Surgical Oncology*,* 26 *(*2*),* 415–424. 10.1245/s10434-018-07110-z; 30569297

[85] Pagès, F., Kirilovsky, A., Mlecnik, B., Asslaber, M., Tosolini, M. et al. (2009). *In situ* cytotoxic and memory T cells predict outcome in patients with early-stage colorectal cancer. Journal of Clinical Oncology*,* 27 *(*35*),* 5944–5951. 10.1200/JCO.2008.19.6147; 19858404

[86] Kwak, Y., Koh, J., Kim, D. W., Kang, S. B., Kim, W. H. et al. (2016). Immunoscore encompassing CD3^+^ and CD8^+^ T cell densities in distant metastasis is a robust prognostic marker for advanced colorectal cancer. Oncotarget*,* 7 *(*49*),* 81778–81790. 10.18632/oncotarget.13207; 27835889 PMC5348429

[87] Galon, J., Mlecnik, B., Bindea, G., Angell, H. K., Berger, A. et al. (2014). Towards the introduction of the ‘Immunoscore’ in the classification of malignant tumours. The Journal of Pathology*,* 232 *(*2*),* 199–209. 10.1002/path.4287; 24122236 PMC4255306

[88] Kim, E. S. (2017). Abemaciclib: First global approval. Drugs*,* 77 *(*18*),* 2063–2070. 10.1007/s40265-017-0840-z; 29128965

[89] Ishii, H., Azuma, K., Kawahara, A., Matsuo, N., Tokito, T. et al. (2017). Programmed cell death-ligand 1 expression and immunoscore in stage II and III non-small cell lung cancer patients receiving adjuvant chemotherapy. Oncotarget*,* 8 *(*37*),* 61618–61625. 10.18632/oncotarget.18651; 28977890 PMC5617450

[90] Ashktorab, H., Ahuja, S., Kannan, L., Llor, X., Ellis, N. A. et al. (2016). A meta-analysis of MSI frequency and race in colorectal cancer. Oncotarget*,* 7 *(*23*),* 34546–34557. 10.18632/oncotarget.8945; 27120810 PMC5085175

[91] Venderbosch, S., Nagtegaal, I. D., Maughan, T. S., Smith, C. G., Cheadle, J. P. et al. (2014). Mismatch repair status and BRAF mutation status in metastatic colorectal cancer patients: A pooled analysis of the CAIRO, CAIRO2, COIN, and FOCUS studies. Clinical Cancer Research*,* 20 *(*20*),* 5322–5330. 10.1158/1078-0432.CCR-14-0332; 25139339 PMC4201568

[92] Koopman, M., Kortman, G. A. M., Mekenkamp, L., Ligtenberg, M. J. L., Hoogerbrugge, N. et al. (2009). Deficient mismatch repair system in patients with sporadic advanced colorectal cancer. British Journal of Cancer*,* 100 *(*2*),* 266–273. 10.1038/sj.bjc.6604867; 19165197 PMC2634718

[93] Popat, S., Hubner, R., Houlston, R. S. (2005). Systematic review of microsatellite instability and colorectal cancer prognosis. Journal of Clinical Oncology*,* 23 *(*3*),* 609–618. 10.1200/JCO.2005.01.086; 15659508

[94] Kim, H., Jen, J., Vogelstein, B., Hamilton, S. R. (1994). Clinical and pathological characteristics of sporadic colorectal carcinomas with DNA replication errors in microsatellite sequences. The American Journal of Pathology*,* 145 *(*1*),* 148–156; 8030745 PMC1887287

[95] Tougeron, D., Sueur, B., Zaanan, A., de la Fouchardiére, C., Sefrioui, D. et al. (2020). Prognosis and chemosensitivity of deficient MMR phenotype in patients with metastatic colorectal cancer: An AGEO retrospective multicenter study. International Journal of Cancer*,* 147 *(*1*),* 285–296. 10.1002/ijc.32879; 31970760

[96] Tougeron, D., Sickersen, G., Mouillet, G., Zaanan, A., Trouilloud, I. et al. (2015). Predictors of disease-free survival in colorectal cancer with microsatellite instability: An AGEO multicentre study. European Journal of Cancer*,* 51 *(*8*),* 925–934. 10.1016/j.ejca.2015.03.011; 25864037

[97] Goldstein, D. B., Tate, S. K., Sisodiya, S. M. (2003). Pharmacogenetics goes genomic. Nature Reviews Genetics*,* 4 *(*12*),* 937–947. 10.1038/nrg1229; 14631354

[98] Des Guetz, G., Uzzan, B., Nicolas, P., Schischmanoff, O., Perret, G. Y. et al. (2009). Microsatellite instability does not predict the efficacy of chemotherapy in metastatic colorectal cancer. A systematic review and meta-analysis. Anticancer Research*,* 29 *(*5*),* 1615–1620; 19443375

[99] Haddad, R., Ogilvie, R. T., Croitoru, M., Muniz, V., Gryfe, R. et al. (2004). Microsatellite instability as a prognostic factor in resected colorectal cancer liver metastases. Annals of Surgical Oncology*,* 11 *(*11*),* 977–982. 10.1245/aso.2004.03.585; 15525826

[100] Barabino, M., Piccolo, G., Tosi, D., Masserano, R., Santambrogio, R. et al. (2020). Correction to: Immunohistochemical evaluation of microsatellite instability in resected colorectal liver metastases: A preliminary experience. Medical Oncology*,* 37 *(*8*),* 71. 10.1007/s12032-020-01399-1; 32715358

[101] de la Fouchardière, C., Cohen, R., Malka, D., Guimbaud, R., Bourien, H. et al. (2019). Characteristics of mutant, deficient mismatch repair/proficient mismatch repair, metastatic colorectal cancer: A multicenter series of 287 patients. The Oncologist*,* 24 *(*12*),* e1331–e1340. 10.1634/theoncologist.2018-0914; 31152084 PMC6975964

[102] Cohen, R., Buhard, O., Cervera, P., Hain, E., Dumont, S. et al. (2017). Clinical and molecular characterisation of hereditary and sporadic metastatic colorectal cancers harbouring microsatellite instability/DNA mismatch repair deficiency. European Journal of Cancer*,* 86*,* 266–274. 10.1016/j.ejca.2017.09.022; 29055842

[103] Goldstein, J., Tran, B., Ensor, J., Gibbs, P., Wong, H. L. et al. (2014). Multicenter retrospective analysis of metastatic colorectal cancer (CRC) with high-level microsatellite instability (MSI-H). Annals of Oncology*,* 25 *(*5*),* 1032–1038. 10.1093/annonc/mdu100; 24585723 PMC4072907

[104] Hagness, M., Foss, A., Line, P. -D., Scholz, T., Jørgensen, P. F. et al. (2013). Liver transplantation for nonresectable liver metastases from colorectal cancer. Annals of Surgery*,* 257 *(*5*),* 800–806. 10.1097/SLA.0b013e3182823957; 23360920

[105] Ahmed, F. A., Kwon, Y. K., Zielsdorf, S., Cooper, J. T., Aziz, H. (2022). Liver transplantation as a curative approach for patients with nonresectable colorectal liver metastases. Experimental and Clinical Transplantation*,* 20 *(*2*),* 113–121. 10.6002/ect.2021.0421; 35282808

[106] Sposito, C., Pietrantonio, F., Maspero, M., Di Benedetto, F., Vivarelli, M. et al. (2023). Improving outcome of selected patients with non-resectable hepatic metastases from colorectal cancer with liver transplantation: A prospective parallel trial (COLT trial). Clinical Colorectal Cancer*,* 22 *(*2*),* 250–255. 10.1016/j.clcc.2023.01.003; 36822922

[107] Gau, L., Ribeiro, M., Pereira, B., Poirot, K., Dupré, A. et al. (2021). Impact of BRAF mutations on clinical outcomes following liver surgery for colorectal liver metastases: An updated meta-analysis. European Journal of Surgical Oncology*,* 47 *(*11*),* 2722–2733. 10.1016/j.ejso.2021.05.039; 34099355

[108] Passiglia, F., Bronte, G., Bazan, V., Galvano, A., Vincenzi, B. et al. (2016). Can KRAS and BRAF mutations limit the benefit of liver resection in metastatic colorectal cancer patients? A systematic review and meta-analysis. Critical Reviews in Oncology/Hematology*,* 99*,* 150–157. 10.1016/j.critrevonc.2015.12.015; 26775732

[109] Brudvik, K. W., Kopetz, S. E., Li, L., Conrad, C., Aloia, T. A. et al. (2015). Meta-analysis of KRAS mutations and survival after resection of colorectal liver metastases. The British Journal of Surgery*,* 102 *(*10*),* 1175–1183. 10.1002/bjs.9870; 26206254

