# Supplementary figures and images for: The challenge of molecular selection in liver-limited metastatic colorectal cancer for surgical resection: a systematic review and meta-analysis in the context of current and future approaches
Source: Oncol Res. 2024 Aug 23;32(9):1407–22. doi: 10.32604/or.2024.049181 (PMC11361904; doi:10.32604/or.2024.049181)

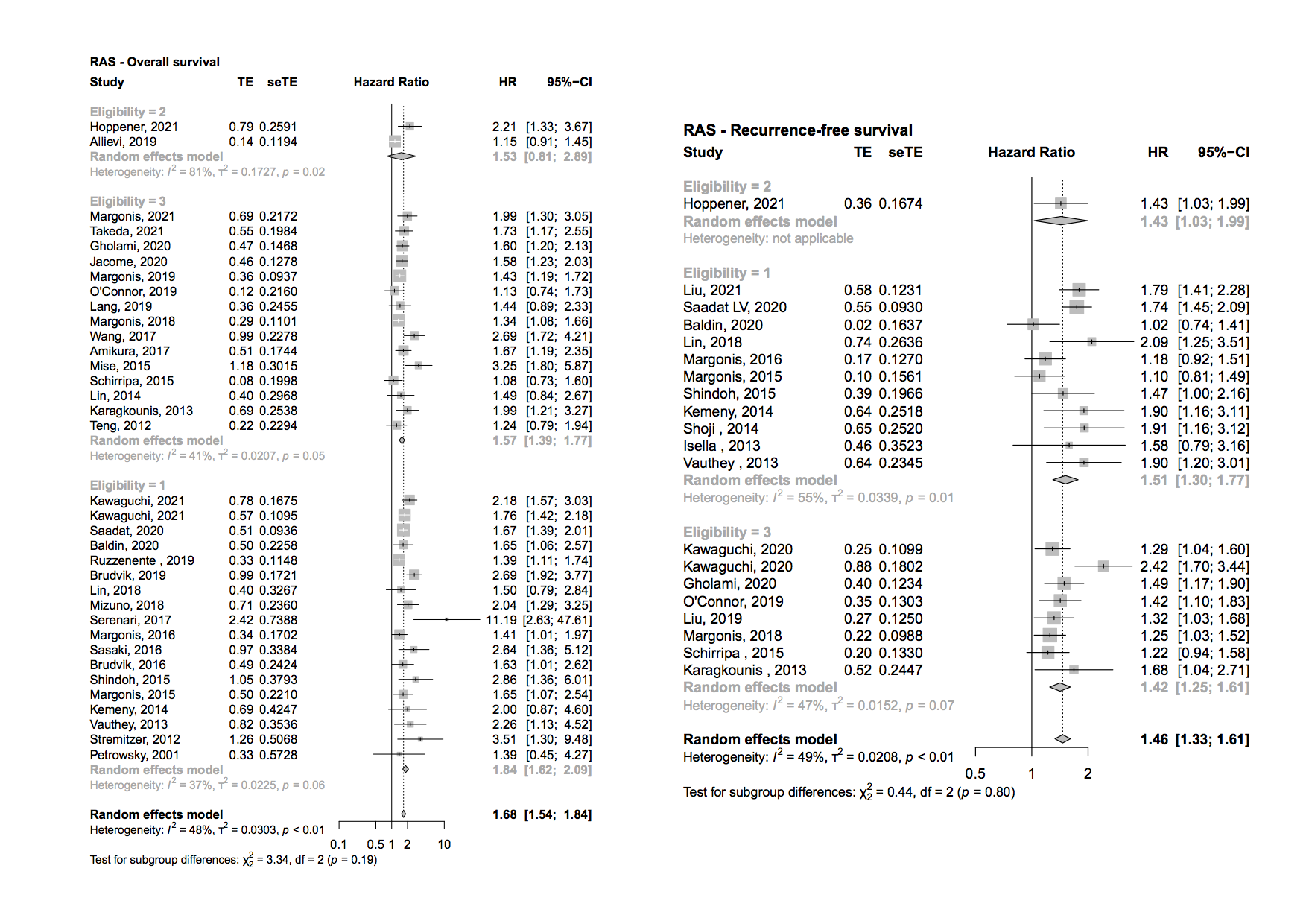

Supplement: Figure S1 [file OncolRes-32-49181-s001.tiff]

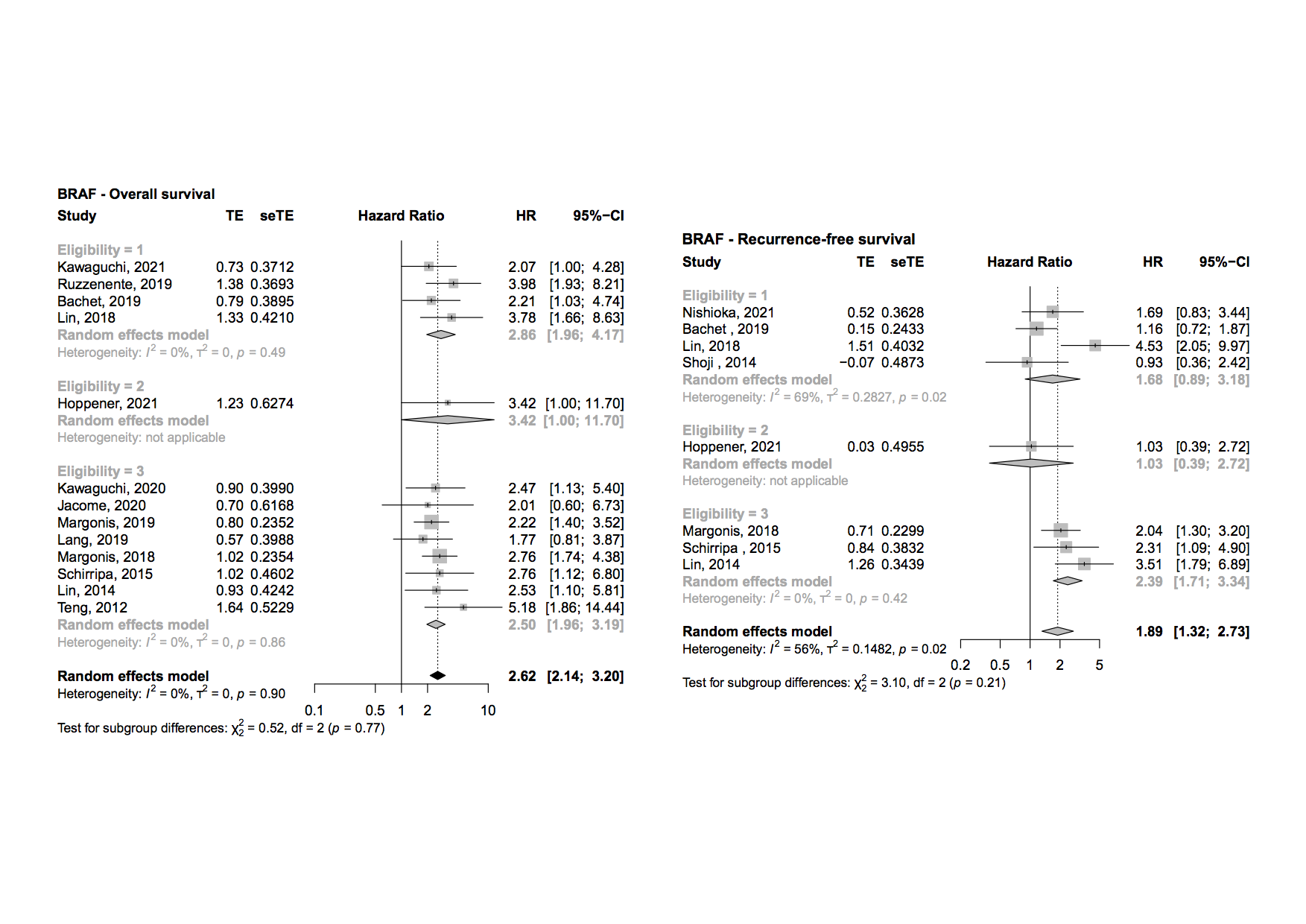

Supplement: Figure S2 [file OncolRes-32-49181-s002.tiff]

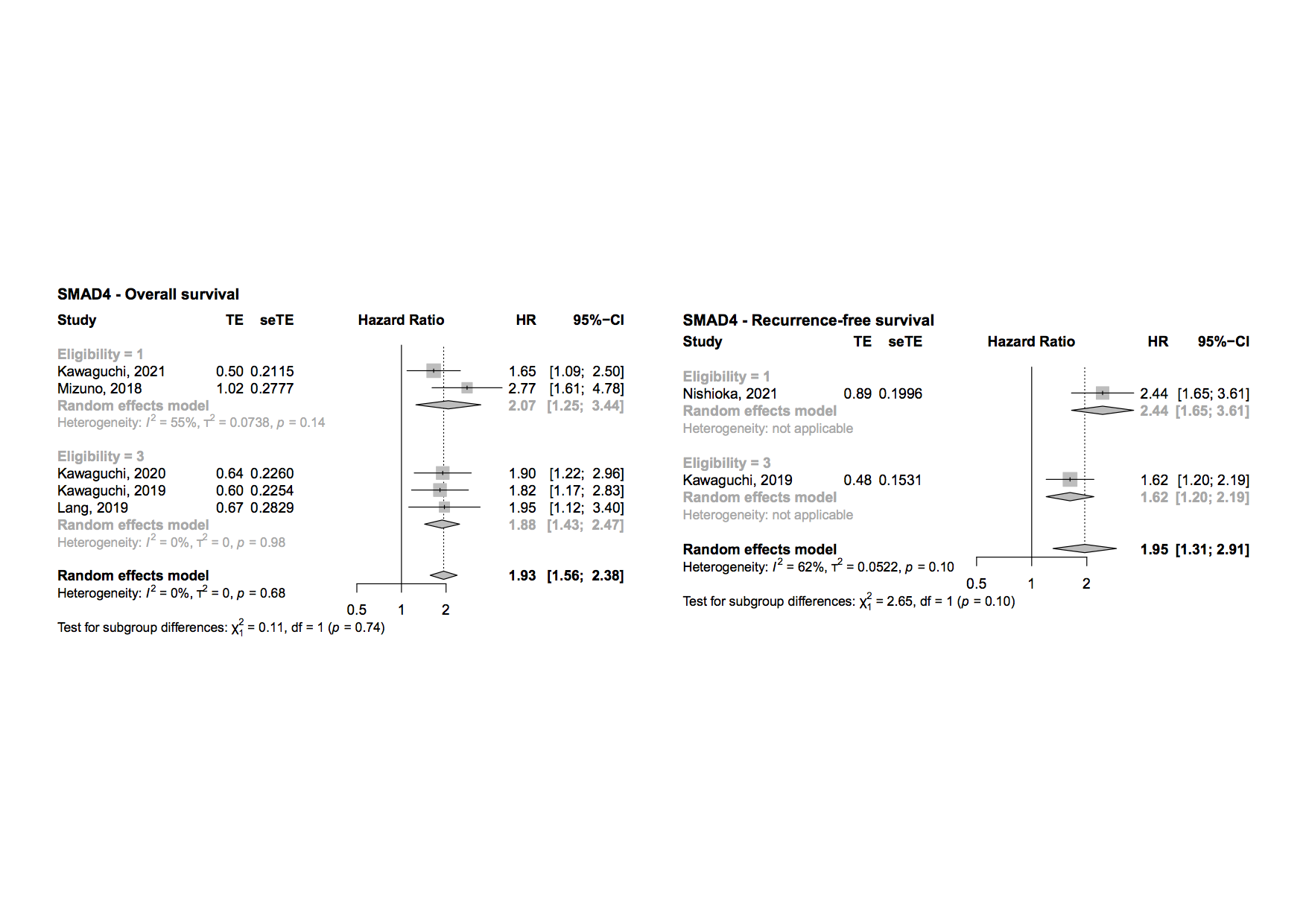

Supplement: Figure S3 [file OncolRes-32-49181-s003.tiff]
